# Measurement of Pharmacokinetics and Tissue Distribution of Four Compounds from *Nauclea officinalis* in Rat Plasma and Tissues through HPLC-MS/MS

**DOI:** 10.1155/2022/5297603

**Published:** 2022-12-21

**Authors:** Yuhuang Wu, Liyan Li, Guxu Ming, Xinyue Ma, Changfu Liang, Yonghui Li, Xiaoning He

**Affiliations:** ^1^Hainan Provincial Key Lab of R&D on Tropic Herbs, School of Pharmacy, Hainan Medical University, Haikou 571199, China; ^2^The Second Affiliated Hospital, Hainan Medical University, Haikou 571199, China

## Abstract

A rapid, sensitive, selective, and accurate HPLC–MS/MS method was developed and validated for the simultaneous determination of chlorogenic acid, naucleactonin C, khaephuoside A 3,4-dimethoxyphenyl-1-O-*β*-apiofuroseyl(1 ⟶ 2)-*β*-D-glucopyranoside in rat plasma and tissues after oral administration of *Nauclea officinalis* extracts. Chloramphenicol was used as an internal standard (IS). The plasma and tissue samples were extracted by protein precipitation with methanol-ethyl acetate (1 : 1, v/v) including 0.1% (v/v) formic acid. The chromatographic separation was achieved by using an C18 column with gradient elution using mobile phase, which consisted of 0.1% formic acid water (A) and acetonitrile (B) and the flow rate of 0.8 mL/min. Mass spectrometric detection was performed in multiple reaction monitoring (MRM) mode utilizing electrospray ionization (ESI) in negative mode. The developed method exhibited good linearity (determination coefficients, *R*^2^ ≥ 0.9849), and the lower limits of quantification were 2, 5, 5, and 25 ng/mL for chlorogenic acid, naucleactonin C, khaephuoside A, and 3,4-dimethoxyphenyl-1-O-*β*-apiofuroseyl(1 ⟶ 2)-*β*-D-glucopyranoside. The intraday and interday precisions (relative standard deviation, RSD) were less than 12.65%, while the accuracy was ranged from 86.31 to 114.17%. The recovery rate were 51.85–97.06%, 75.99–106.68%, 77.46–105.35%, and 68.36–103.75% for chlorogenic acid, naucleactonin C, khaephuoside A, and 3,4-dimethoxyphenyl-1-O-*β*-apiofuroseyl(1 ⟶ 2)-*β*-D-glucopyranoside the matrix effects were 50.17–116.62%, 86.75–115.99%, 45.79–87.44%, and 51.60–92.34% for chlorogenic acid, naucleactonin C, khaephuoside A, and 3,4-dimethoxyphenyl-1-O-*β*-apiofuroseyl(1 ⟶ 2)-*β*-D-glucopyranoside in different matrix. The developed method was successfully applied to a pharmacokinetic study and tissue distribution of four compounds in rats after oral administration of *Nauclea officinalis* extracts.

## 1. Introduction

*Nauclea officinalis* Pierre. ex Pitard, is one of the most commonly used traditional medicines in China and is mainly distributed in Hainan, Guangxi, Guangdong, and other provinces in the south of China [1]. Modern pharmacological studies reported that *Nauclea officinalis* exhibits various biological properties such as antibacterial, anti-inflammatory, and analgesic activity [2–6]. As a traditional Chinese medicine, the stems and twigs of *Nauclea officinalis* are used for the treatment of colds, fever, acute tonsillitis, sore throat, and other diseases [1, 7]. A number of different kinds of compounds have been isolated and identified from *Nauclea officinalis*, including alkaloids, phenolic acids, iridoids, pentacyclic triterpenoids, and flavonoids [8–12]. Previous studies showed that alkaloids were the main bioactive compound in *Nauclea officinalis* [13–15]. Recent literature reported that phenolic compounds such as protocatechuic acid and chlorogenic acid possess antioxidant and antibacterial effects [16–18]. Therefore, the phenolic compounds may be another kind of active ingredient in *Nauclea officinalis.*

As a valuable work, the pharmacokinetics research could not only explain the absorption, distribution, metabolism, and excretion of bioactive compounds but also reveal the mechanism of action and the cause of toxicity [19]. Several reports have been published on the pharmacokinetics of the alkaloids of *Nauclea officinalis.* For example, the pharmacokinetics of alkaloids of *Nauclea officinalis* extracts and Danmu preparations in rat plasma [20–22] and the pharmacokinetics of Strictosamide in dog plasma [23]. However, as one of the main active compounds of *Nauclea officinalis*, the pharmacokinetics of phenolic acid compounds were rarely researched. To our knowledge, the quantitative detection of pharmacokinetics and tissue distribution of chlorogenic acid, naucleactonin C, khaephuoside A, and 3,4-dimethoxyphenyl-1-O-*β*-apiofuroseyl(1 ⟶ 2)-*β*-D-glucopyranoside of *Nauclea officinalis* extract have not been reported in rats. Hence, it is necessary to establish rapid and accurate approaches to investigate its pharmacokinetics and tissue distribution.

The aim of the present investigation is to develop a reliable and sensitive method based on HPLC-MS/MS to quantify the four compounds of *Nauclea officinalis* extract in the plasma and tissue distribution of rats. It is expected that the results of this study can provide a useful reference for understanding the mechanism of action, safety evaluation, and clinical application of *Nauclea officinalis*.

## 2. Material and Methods

### 2.1. Chemicals and Reagents

The stems of *Nauclea officinalis* were collected from Qiongzhong County, Hainan Province, and identified by Prof. Jianping Tian of Hainan Medical University. Standards of naucleactonin C, khaephuoside A, and 3,4-dimethoxyphenyl-1-O-*β*-apiofuroseyl(1 ⟶ 2)-*β*-D-glucopyranoside were isolated from *Nauclea officinalis* in our laboratory by silica gel column, semi-preparative high performance liquid chromatography, which structure was identified by NMR, and the HPLC purity was over than 98%. The standards for chlorogenic acid were purchased from Chengdu Pufeide Biological Technology Co., Ltd (HPLC >98%, Sichuan, China). Chloramphenicol was provided by the National Institute for Food and Drug Control (Beijing, China). Methanol and acetonitrile were all chromatographically pure and purchased from Fisher Scientific (Fair Lawn, NJ, USA); ethyl acetate and formic acid were all chromatographically pure and purchased from Aladdin Industrial Corporation (Shanghai, China); purified water was prepared by a LabTower EDI system (Thermo Scientific, USA).

### 2.2. Instrument and Analytical Conditions

Chromatographic analysis was performed on the LC-20ADXR System (Shimadzu, Japan). The Phenomenex Kinete EVO C18 100 Å (50 mm × 2.1 mm, 5 *μ*m) column was used as the analytical column maintained at 40°C with (A) 0.1% formic acid aqueous solution and (B) acetonitrile as the mobile phase. The gradient elution program was operated as follows: 2% B (0–0.5 min), 2–25% B (0.5–0.51 min), 25-55% B (0.51–3.0 min), 55-95% B (3.0–3.01 min), 95% B (3.01–5.0 min), 95–2% B (5.0–5.01 min), and 2% B (5.01–6.0 min). The flow rate was 0.8 mL/min, and the injection volume was 5 *μ*L.

Mass spectrometric detection was performed on an AB-SCIEX API 4000 mass spectrometer (AB SCIEX, Singapore) with ESI in negative ion multiple reaction monitoring (MRM) mode. The optimized instrument parameters were as follows: nebulizer gas: 50 psi, heated by N_2_ gas: 55 psi, ion spray voltage: −4500 V and temperature: 550°C; nebulizer, blowback gas, and collision gas were nitrogen; declustering potential (DP), collision energy (CE), and collision cell exit potential (CXP) of the four analytes and chloramphenicol (IS) are shown in Table 1.

### 2.3. Preparation of *Nauclea officinalis* Extract

Powdered *Nauclea officinalis* stems (1 kg) were accurately weighed and heated and refluxed by water (1 : 10, w/v) for 2 h. The extractions were combined and concentrated under reduced pressure to obtain the 25 g of crude water extracts.

### 2.4. Preparations of Standard Solutions, IS, and Quality Control (QC) Samples

The chlorogenic acid, naucleactonin C, khaephuoside A, and 3,4-dimethoxyphenyl-1-O-*β*-apiofuroseyl(1 ⟶ 2)-*β*-D-glucopyranoside and chloramphenicol (IS) were dissolved in methanol at a concentration of 1 mg/mL as the stock solution. A series of mixed standard working solutions were prepared by diluting the primary mixed stock solution with methanol at appropriate ratios. All the solutions were kept away from light at 4°C until analysis.

The calibration standards were prepared by spiking 10 *μ*L of the mixed standard solution and 10 *μ*L (500 ng/mL) IS solution into 90 *μ*L rat blank matrixes at final concentrations of 2–2400 ng/mL of chlorogenic acid, 5–2400 ng/mL of naucleactonin C, 5-2000 ng/mL of khaephuoside A, and 25–2400 ng/mL of 3,4-dimethoxyphenyl-1-O-*β*-apiofuroseyl(1 ⟶ 2)-*β*-D-glucopyranoside.

The QC samples were prepared at concentrations of 2, 5, 100, and 2400 ng/mL for chlorogenic acid; 5, 8, 140, and 2400 ng/mL for naucleactonin C; 5, 8, 120, and 2000 ng/mL for khaephuoside A; 25, 50, 350, and 2400 ng/mL for 3,4-dimethoxyphenyl-1-O-*β*-apiofuroseyl(1 ⟶ 2)-*β*-D-glucopyranoside, respectively. The IS concentration was 45 ng/mL in all calibration standards and QC.

### 2.5. Animals

Male Sprague–Dawley (SD) rats (250 ± 20 g weight) were purchased from Tianqin Biotechnology Co. Ltd. (License No. 4307256220100013061), Changsha, China. The animals were maintained at 22 ± 2°C and 60% ± 10% humidity with a 12 h light/dark cycle and allowed free access to food and water. All animal experiments were performed in accordance with the Institutional Animal Care and Use Committee at the Hainan Medical University (Haikou, China).

### 2.6. Preparation of Biological Samples

An aliquot of 100 *μ*L of rat plasma and tissue homogenate were transferred to a 2 mL tube. Subsequently, 10 *μ*L IS (500 ng/mL) were added followed by 1 mL of methanol-ethyl acetate (1 : 1, v/v) including 0.1% (v/v) formic acid. The sample was then vortexed for 1 min and centrifuged at 13,000 rpm for 5 min. Thereafter, the supernatant was dried at 37°C with nitrogen, which the residue was reconstituted with 80 *μ*L methanol, centrifuged at 13,000 rpm for 5 min, and 5 *μ*L of the sample were injected into the HPLC-MS/MS system for analysis.

### 2.7. Method Validation

The method validation assays were carried out according to the U.S. Food and Drug Administration (FDA) Bioanalytical Method Validation (Food and Drug Administration, 2018), including specificity, linearity, recovery, matrix effect, precision, accuracy, and stability [24].

### 2.8. Pharmacokinetic Study

Six male SD rats were tested in pharmacokinetics studies. The rats were housed at 22 ± 2°C and fasted for 12 h with free access to water prior to dosing, which were orally administered *Nauclea officinalis* extracts at the dose of 2 g/kg (equivalent to 32.74 mg/kg chlorogenic acid, 0.78 mg/kg naucleactonin C, 14.1 mg/kg khaephuoside A, and 10.39 mg/kg 3,4-dimethoxyphenyl-1-O-*β*-apiofuroseyl(1 ⟶ 2)-*β*-D-glucopyranoside). The blood samples (approximately 0.5 mL) were collected via the venous plexus of the eye socket at 0.083, 0.25, 0.5, 1, 2, 4, 6, 8, 1, 12, and 24 h under anesthesia. Next, each sample was immediately centrifuged at 4000 rpm for 10 min to acquire the plasma. The plasma was transferred to new tubes and stored at −80°C until further use. The pharmacokinetics parameters of the four compounds in rat plasma were calculated by the noncompartment model using Drug and Statistics (DAS 3.3.0) software (Beijing, China).

### 2.9. Tissue Distribution

Thirty rats were divided into five groups (*n* = 6) at random, which were orally administered *Nauclea officinalis* extracts at a dose of 2 g/kg for tissue distribution conducted at 0.5, 1, 2, 4, and 6 h. During the collection, the heart, liver, spleen, lung, kidney, stomach, small intestine, and brain were rinsed with physiological saline solution to get rid of the blood or content and blotted on filter paper and then weighed. Each tissue sample was accurately weighed and homogenized by using physiological saline at four times the tissue weight (w/v). The homogenates were stored at −80°C until analysis.

## 3. Results and Discussion

### 3.1. Optimization of Chromatographic and Mass Spectrometric Conditions

Optimization of chromatographic conditions: by analyzing the separation effect and peak shape of four analytes and the IS on different columns, we found that the Phenomenex Kinete EVO C18 column gave the best separation and peak shape. Subsequently, by exploring different mobile phase systems, such as methanol-water and acetonitrile-water, we found that the four analytes had better response values in acetonitrile. Finally, by assessing the pH of the mobile phase (0.1, 0.2, 0.5, and 0.8% formic acid solution), the response values of four analytes were highest when the concentration of formic acid was 0.1%. Therefore, 0.1% formic acid water-acetonitrile was selected for use as the mobile phase.

**Figure 1:**
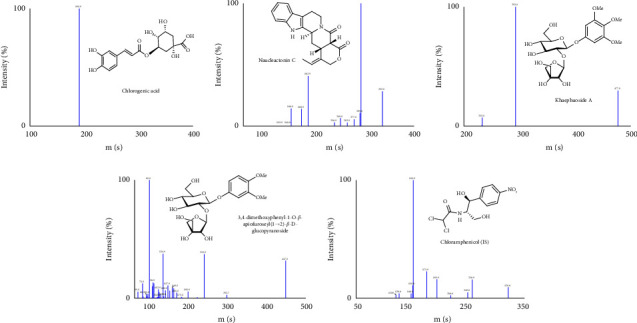
Chemical structures, precursor ion, and product ion mass spectra of four analytes and IS.

Optimization of mass spectrometric conditions: the four analytes and the IS had strong [M-H]^−^ peaks in the negative ion mode, which can be easily broken and used for stable fragment analysis and detection. Therefore, the ESI in negative ion mode and Q1, MS2, and MRM scan modes were adopted. The chemical structures, precursor ion, and product ion of four analytes and IS are shown in Figure 1.

### 3.2. Optimization of the Sample Preparation

For sample processing, a simple protein precipitation method was first tried for methanol and acetonitrile, respectively, but the recoveries of chlorogenic acid and naucleactonin C were unsatisfactory. In addition, liquid-liquid extraction (LLE) was tried for various solvents tested, including n-butanol, dichloromethane, isopropanol, methyl tert-butyl ether, and ethyl acetate, but no satisfactory recovery was obtained for khaephuoside A, and 3,4-dimethoxyphenyl-1-O-*β*-apiofuroseyl(1 ⟶ 2)-*β*-D-glucopyranoside. The extraction of chlorogenic acid should be in an acidic condition. Therefore, the extraction results of chlorogenic acid at different pH are investigated.

Finally, the combination method using mixture solution of methanol-ethyl acetate (1 : 1, v/v) including 0.1% (v/v) formic acid was selected. Consequently, satisfactory and consistent recovery from plasma and tissues samples was achieved for four analytes and IS.

### 3.3. Method Validation

#### 3.3.1. Selectivity

**Figure 2:**
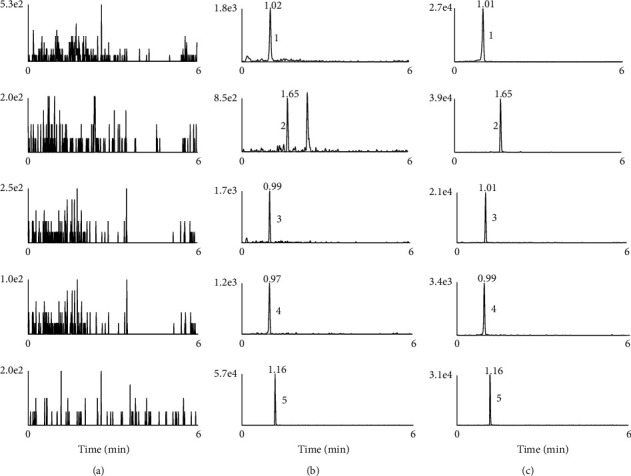
Typical chromatograms of four analyte and IS in rat plasma: (a) blank plasma samples, (b) blank plasma samples spiked with four analytes (LLOQ) and IS, (c) rat plasma samples at 0.5 h after oral administration the *Nauclea officinalis* extracts spiked with IS; (1) chlorogenic acid, (2) naucleactonin C, (3) khaephuoside A, (4) 3,4-dimethoxyphenyl-1-O-*β*-apiofuroseyl(1 ⟶ 2)-*β*-D-glucopyranoside, (5) chloramphenicol (IS).

The selectivity was assessed by analyzing chromatograms of blank rat plasma and tissue homogenates from different batches, blank plasma and tissue homogenates spiked with analytes and IS, and plasma, tissue samples obtained from rats after oral administration of *Nauclea officinalis* extracts (*n* = 6).  Figure 2 shows typical chromatograms of four analytes and IS in rat plasma (Figures S1 and S2 of the Supporting Material show typical chromatograms of four analytes and IS in liver tissue and kidney tissue). Under the given condition, Chlorogenic acid, Naucleactonin C, Khaephuoside A, 3,4-dimethoxyphenyl-1-O-*β*-apiofuroseyl(1 ⟶ 2)-*β*-D-glucopyranoside, and IS were eluted approximately at retention time of 1.02 min, 1.65 min, 0.99 min, 0.97 min, and 1.16 min, respectively. No interfering endogenous substances were found at the respective retention times.

#### 3.3.2. Linearity of Calibration Curve and Lower Limit of Quantification

The calibration curves were constructed by plotting the peak areas ratios of chlorogenic acid/IS, Naucleactonin C/IS, Khaephuoside A/IS, and 3,4-dimethoxyphenyl-1-O-*β*-apiofuroseyl(1 ⟶ 2)-*β*-D-glucopyranoside/IS versus theoretical concentrations. This method exhibited a good linear response for the range of concentrations from 2 to 2500 ng/mL in plasma and from 2 to 2500 ng/mL in tissues; all determination coefficients (*R*^2^) were greater than 0.9849. The lower limit of quantification (LLOQ) was defined as the lowest concentration of a signal-noise (S/N) ratios of 10 : 1, respectively, the precision was less than 15% and the accuracy was within ±20%. The limits were adequate for studies of pharmacokinetics and tissue distribution by oral administration of *Nauclea officinalis* extracts. Data from the determination are shown in Table 2.

#### 3.3.3. Accuracy and Precision

Accuracy and precision were evaluated by analyzing QC samples at high, medium, low, and LLOQ concentrations (*n* = 6) on the same day and three consecutive days using the standard curve, respectively. The acceptable limits of accuracy were required to be within ±15% of the actual value except when at LLOQ, and the intra- and interday precision and accuracy data of the determination were shown in Table 3. In our study, the intraday precision ranged from 0.62 to 12.65%, and intraday accuracy ranged from 86.48 to 114.17%. The interday precision ranged from 2.42 to 12.61%, and the interday accuracy ranged from 86.31 to 113.99%. The results showed the accuracy and precision were within the acceptable limits, which proved the method was reproducible, reliable, and accurate for the determination of four analytes in rat plasma and tissue samples.

#### 3.3.4. Extraction Recovery and Matrix Effect

Six batches of blank plasma from independent sources were used to obtain extracted samples, postextracted spiked samples, and unextracted samples at high, medium, low, and LLOQ concentrations. The peak areas of the three types of samples were recorded as A, B, and C, respectively. Extraction recovery was evaluated by A/B. Matrix effects were calculated by comparing the A/C of analytes. The extraction, recovery, and matrix effect of four analytes in rat plasma and tissues are shown in Table 4. The recovery rate were 51.85–97.06%, 75.99–106.68%, 77.46–105.35%, and 68.36–103.75% for chlorogenic acid, naucleactonin C, khaephuoside A, and 3,4-dimethoxyphenyl-1-O-*β*-apiofuroseyl(1 ⟶ 2)-*β*-D-glucopyranoside and the matrix effects were 50.17–116.62%, 86.75–115.99%, 45.79–87.44%, and 51.60–92.34% for chlorogenic acid, naucleactonin C, khaephuoside A, and 3,4-dimethoxyphenyl-1-O-*β*-apiofuroseyl(1 ⟶ 2)-*β*-D-glucopyranoside in different matrices, indicating some ion inhibitory effects for four compounds in the biological samples, the RSD (relative standard deviation) of recovery and matrix effect were less than 15%. There were no significant differences in recoveries and matrix effects between the four compounds at different concentrations in the same matrix.

#### 3.3.5. Stability

The stability was evaluated by analyzing QC samples at high, medium, low, and LLOQ concentrations (*n* = 6) under different storage conditions, including room temperature for 4 h, auto-sampler (4°C) for 8 h, three freeze-thaw cycles (freeze cycle was at −20°C while thaw cycle was done at room temperature) and −80°C for 30 days. The results are shown in Table 5. It was evident from these data that four storage conditions have no significant effect on the sample. The results indicated that four analytes were stable under these four storage conditions.

### 3.4. Pharmacokinetic Study

**Figure 3:**
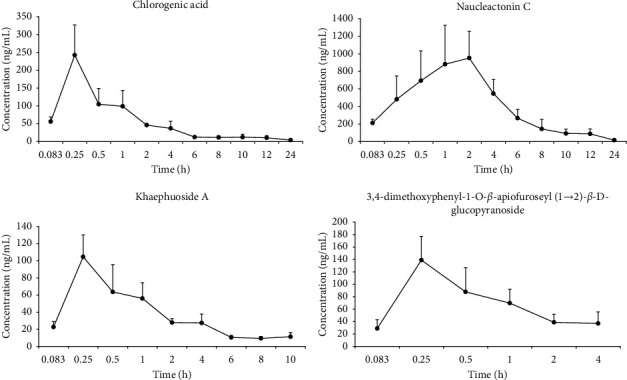
Concentration-time profiles of four analytes in rat plasma following oral administration of *Nauclea officinalis* extracts at a dose of 2 g/kg.

The validated analytical method was successfully applied to the quantitative estimation of chlorogenic acid, naucleactonin C, khaephuoside A, and 3,4-dimethoxyphenyl-1-O-*β*-apiofuroseyl(1 ⟶ 2)-*β*-D-glucopyranoside in the plasma samples following oral administration of *Nauclea officinalis* extract at a dosage of 2 g/kg. Pharmacokinetic parameters of four compounds were calculated by noncompartmental model using DAS 3.3.0 software. The mean plasma concentration–time-curve of four analytes are shown in Figure 3, and the main pharmacokinetic parameters were estimated of four analytes in Table 6.

These compounds were detected at 5 min after oral administration of *Nauclea officinalis* extracts, indicating four compounds were rapidly absorbed, exhibiting a therapeutic effect. Chlorogenic acid, khaephuoside A, and 3,4-dimethoxyphenyl-1-O-*β*-apiofuroseyl(1 ⟶ 2)-*β*-D-glucopyranoside were rapidly absorbed and peaked at approximately 0.5 h, while naucleactonin C peaked at 1.66 h, indicating that naucleactonin C was absorbed relatively slowly. The content of naucleactonin C was much lower than that of the other three compounds in the *Nauclea officinalis* extracts, but naucleactonin C was present in a much larger amount than other three compounds for AUC_0−*t*_ (area under the plasma concentration–time curve) and AUC_0−∞_, indicating that naucleactonin C may had excellent bioavailability. However, the bioavailability of chlorogenic acid and khaephuoside A may not be satisfactory, the CL of chlorogenic acid and khaephuoside A were 62.93 ± 15.996 and 54.40 ± 6.04 L/h/kg after oral administration of *Nauclea officinalis* extract, respectively. Excessive clearance rate will affect the residence time of the drug in the body, thereby reducing the efficacy. In addition, literature reported that the prototype chlorogenic acid absorbed into the blood by oral chlorogenic acid accounts for only 30% [25], which greatly affected the pharmacological activity of chlorogenic acid from *Nauclea officinalis*. Therefore, considering the clinical application of *Nauclea officinalis* [26], we could design *Nauclea officinalis* to different dosage forms with advanced technology in order to improve the bioavailability and finally increase the efficacy.

### 3.5. Tissue Distribution Study

**Figure 4:**
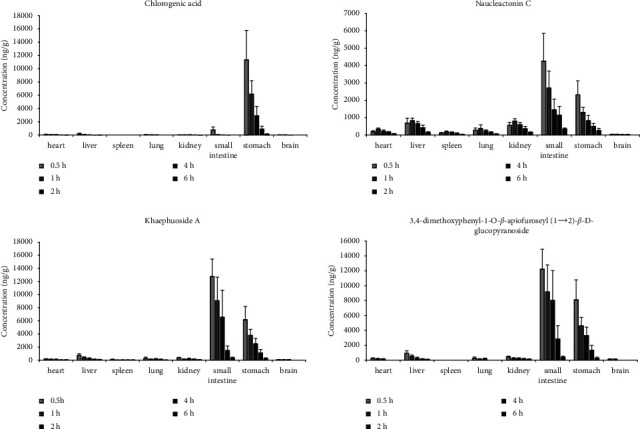
Mean concentration of four analytes in the heart, liver, spleen, lung, kidney, small intestine, stomach, and brain at 0.5, 1, 2, 4, and 6 h after oral administration of *Nauclea officinalis* extracts at a dose of 2 g/kg.

This method was also applied to investigate the tissue distribution of chlorogenic acid, naucleactonin C, khaephuoside A, and 3,4-dimethoxyphenyl-1-O-*β*-apiofuroseyl(1 ⟶ 2)-*β*-D-glucopyranoside in rats. The result is shown in Figure 4 that naucleactonin C and khaephuoside A could be detected in all studied tissue. However, chlorogenic acid and 3,4-dimethoxyphenyl-1-O-*β*-apiofuroseyl(1 ⟶ 2)-*β*-D-glucopyranoside were not detected in the spleen. The concentration orders in eight different tissues were ranked as 3,4-dimethoxyphenyl-1-O-*β*-apiofuroseyl(1 ⟶ 2)-*β-*D-glucopyranoside > Khaephuoside A > Naucleactonin C > Chlorogenic acid in the small intestine, stomach, heart, liver, spleen, lung, and brain tissues, and naucleactonin C > 3,4-dimethoxyphenyl-1-O-*β*-apiofuroseyl(1 ⟶ 2)-*β*-D-glucopyranoside > Khaephuoside A > Chlorogenic acid in the kidney. The contents of the four compounds in the liver, kidney, and lung were relatively high within after oral administration of *Nauclea officinalis* extrac, indicating that the liver, kidney, and lung may be the main target organs for the pharmacological effects of *Nauclea officinalis*. This result is consistent with existing research on the inhibition of bronchitis by *Nauclea officinalis* [27]. The results show that all compounds except chlorogenic acid were more abundant in the small intestine than in the stomach, indicating that most of naucleactonin C, khaephuoside A, and 3,4-dimethoxyphenyl-1-O-*β*-apiofuroseyl(1 ⟶ 2)-*β*-D-glucopyranoside possibly absorbed by the small intestine after oral administration of *Nauclea officinalis* extract for enter the systemic circulation system. With the exception of chlorogenic acid, the peak times and concentrations of the other three compounds in most tissues are nearly identical to those in plasma, which means that the distribution of the other three compounds depends on the blood flow or perfusion rate of the organ [28]. The peak concentration of chlorogenic acid in most tissues is much lower than in plasma and may be related to the absorption of the compound in the tissue. Drugs enter tissues that receive high blood flow first, followed by those that receive low blood flow [29]. Our study demonstrated that the four compounds are mainly distributed in organs with relatively large blood flows, such as the liver and kidneys. In addition to this, the first-pass effect of oral administration might be the main cause of high distribution in the liver.

## 4. Conclusion

We report the development and validation of a sensitive, rapid, and reliable HPLC–MS/MS analytical method for the simultaneous determination and quantification of chlorogenic acid, naucleactonin C, khaephuoside A, and 3,4-dimethoxyphenyl-1-O-*β*-apiofuroseyl(1 ⟶ 2)-*β*-D-glucopyranoside in the plasma and tissues of rats. This validated analytical method was assessed on the basis of the FDA guidelines for bioanalytical method validation and applied the study of pharmacokinetics and tissue distribution of oral administration of *Nauclea officinalis* extract in SD rats. To the best of our knowledge, this is the first study that determined the pharmacokinetic and tissue distribution of naucleactonin C, khaephuoside A, and 3,4-dimethoxyphenyl-1-O-*β*-apiofuroseyl(1 ⟶ 2)-*β*-D-glucopyranoside. In addition, the pharmacokinetics and tissue distribution of chlorogenic acid after oral administration of *Nauclea officinalis* extract in rats was also reported for the first time.

## Figures and Tables

**Table 1:** Optimized multiple reaction monitoring parameters for the detection of four analytes and IS.

Compounds	Retention time (min)	Precursor ion (*m*/*z*)	Quantitative ion (*m*/*z*)	DP (V)	CE (V)	CXP (V)
Chlorogenic acid	1.02	353.0	190.9	−51	−22.9	−10.5
Naucleactonin C	1.65	335.1	182.9	−110	−37	−9
Khaephuoside A	0.99	477.1	293.0	−98	−18	−6
3,4-Dimethoxyphenyl-1-O-*β*-apiofuroseyl(1 ⟶ 2)-*β*-D-glucopyranoside	0.97	447.1	232.9	−84	−25	−14
Chloramphenicol (IS)	1.16	320.8	175.9	−85	−22	−9

Abbreviations. DP, declustering potential; CE, collision energy; CXP, collision cell exit potential.

**Table 2:** Summary of standard curves, correlation coefficients, LLOQ, and linear ranges of four analytes in plasma and tissue samples.

Compounds	Matrix	Linear regression equation	*R * ^2^	LLOQ (ng/mL)	Linear range (ng/mL)
Chlorogenic acid	Plasma	*y* = 0.0288*x* + 0.00193	0.9994	2	2–2500
	Heart	*y* = 0.0063*x* − 0.00771	0.9849	2	2–2500
	Liver	*y* = 0.0267*x* − 0.0323	0.9980	2	2–2500
	Spleen	*y* = 0.0392*x* + 0.0277	0.9886	2	2–2500
	Lung	*y* = 0.0442*x* + 0.0239	0.9958	2	2–2500
	Kidney	*y* = 0.0217*x* − 0.0302	0.9950	2	2–2500
	Small intestine	*y* = 0.0334*x* − 0.0401	0.9964	2	2–2500
	Stomach	*y* = 0.0402*x* − 0.0129	0.9952	2	2–2500
	Brain	*y* = 0.0499*x* − 0.0562	0.9960	2	2–2500

Naucleactonin C	Plasma	*y* = 0.00535*x* + 0.0207	0.9964	5	5–2500
	Heart	*y* = 0.00313*x* − 0.00116	0.9926	5	5–2500
	Liver	*y* = 0.00395*x* − 0.0101	0.9964	5	5–2500
	Spleen	*y* = 0.00511*x* − 0.00526	0.9950	5	5–2500
	Lung	*y* = 0.00534*x* − 0.00263	0.9980	5	5–2500
	Kidney	*y* = 0.00363*x* − 0.0098	0.9960	5	5–2500
	Small intestine	*y* = 0.00224*x* − 0.00527	0.9968	5	5–2500
	Stomach	*y* = 0.00465*x* − 0.0138	0.9972	5	5–2500
	Brain	*y* = 0.00552*x* − 0.0163	0.9932	5	5–2500

Khaephuoside A	Plasma	*y* = 0.00316*x* + 0.0153	0.9954	5	5–2000
	Heart	*y* = 0.00673*x* − 0.005	0.9946	5	5–2000
	Liver	*y* = 0.0063*x* − 0.00502	0.9962	5	5–2000
	Spleen	*y* = 0.011*x* − 0.0054	0.9980	5	5–2000
	Lung	*y* = 0.0099*x* − 0.00166	0.9978	5	5–2000
	Kidney	*y* = 0.00531*x* − 0.0047	0.9954	5	5–2000
	Small intestine	*y* = 0.0058*x* − 0.0156	0.9906	5	5–2000
	Stomach	*y* = 0.00745*x* − 0.00837	0.9920	5	5–2000
	Brain	*y* = 0.0126*x* + 0.000377	0.9938	5	5–2000

3,4-Dimethoxyphenyl-1-O-*β*-apiofuroseyl(1 ⟶ 2)-*β*-D-glucopyranoside	Plasma	*y* = 0.000845*x* + 0.00396	0.9984	25	25–2500
	Heart	*y* = 0.000804*x* + 0.00159	0.9849	25	25–2500
	Liver	*y* = 0.000873*x* − 0.00515	0.9968	25	25–2500
	Spleen	*y* = 0.00134*x* + 0.00947	0.9894	25	25–2500
	Lung	*y* = 0.00138*x* + 0.00626	0.9964	25	25–2500
	Kidney	*y* = 0.000633*x* − 0.000538	0.9930	25	25–2500
	Small intestine	*y* = 0.00109*x* + 0.000966	0.9962	25	25–2500
	Stomach	*y* = 0.00119*x* + 0.00549	0.9964	25	25–2500
	Brain	*y* = 0.00163*x* − 0.0149	0.9952	25	25–2500

Abbreviations. LLOQ, lower limit of quantification; *R*^2^, determination coefficients.

**Table 3:** Summary of intraday and interday precisions and accuracies of four analytes in plasma and tissue samples.

Compounds	Matrix	Concentration (ng/mL)	Intraday (*n* = 6)		Interday (*n* = 18)	
			Accuracy	RSD (%)	Accuracy	RSD (%)
Chlorogenic acid	Plasma	2	104.02 ± 11.91	11.45	100.64 ± 11.67	11.60
		4	97.72 ± 9.23	9.45	96.09 ± 7.08	7.36
		100	104.42 ± 6.23	5.97	104.29 ± 10.6	10.16
		2400	101.22 ± 3.20	3.16	96.59 ± 8.11	8.40
	Heart	2	101.90 ± 12.14	11.92	109.34 ± 11.74	10.74
		4	99.35 ± 3.89	3.91	93.67 ± 6.13	6.54
		100	96.57 ± 4.81	4.98	94.46 ± 7.80	8.26
		2400	109.00 ± 5.90	5.41	102.88 ± 6.85	6.66
	Liver	2	101.65 ± 2.82	2.78	100.88 ± 5.38	5.33
		4	101.45 ± 2.98	2.93	98.59 ± 6.72	6.82
		100	100.30 ± 2.89	2.88	97.78 ± 4.89	5.00
		2400	95.07 ± 5.74	6.04	97.11 ± 5.58	5.75
	Spleen	2	96.40 ± 7.63	7.92	102.14 ± 8.90	8.71
		4	95.40 ± 4.20	4.40	101.28 ± 9.61	9.49
		100	102.40 ± 2.00	1.95	111.52 ± 7.00	6.27
		2400	94.93 ± 4.81	5.07	108.26 ± 10.41	9.62
	Lung	2	101.53 ± 6.40	6.30	101.22 ± 7.45	7.36
		4	99.75 ± 6.09	6.10	93.33 ± 11.17	11.97
		100	108.33 ± 5.47	5.04	111.33 ± 6.49	5.83
		2400	100.60 ± 3.35	3.33	101.63 ± 5.07	4.99
	Kidney	2	98.12 ± 5.36	5.47	96.23 ± 5.33	5.54
		4	100.13 ± 2.17	2.17	92.09 ± 7.21	7.83
		100	99.37 ± 5.20	5.23	96.10 ± 6.36	6.62
		2400	104.47 ± 5.79	5.54	99.47 ± 6.06	6.10
	Small intestine	2	93.27 ± 4.13	4.43	96.03 ± 7.86	8.19
		4	86.48 ± 5.09	5.89	93.40 ± 10.75	11.51
		100	89.75 ± 7.96	8.87	96.61 ± 10.06	10.41
		2400	92.70 ± 6.63	7.16	93.01 ± 5.27	5.66
	Stomach	2	105.87 ± 7.87	7.43	107.84 ± 7.45	6.90
		4	93.17 ± 7.02	7.53	98.76 ± 6.44	6.52
		100	106.00 ± 3.95	3.73	106.35 ± 4.46	4.19
		2400	100.63 ± 4.18	4.15	99.41 ± 4.81	4.84
	Brain	2	109.27 ± 6.74	6.17	104.39 ± 9.47	9.07
		4	101.00 ± 3.81	3.77	101.29 ± 5.42	5.35
		100	106.00 ± 4.94	4.66	106.77 ± 7.16	6.70
		2400	101.27 ± 2.25	2.22	105.62 ± 7.04	6.67

Naucleactonin C	Plasma	5	95.62 ± 12.10	12.65	98.24 ± 10.30	10.48
		8	100.53 ± 11.78	11.71	96.43 ± 12.16	12.61
		140	99.95 ± 6.49	6.50	95.76 ± 7.18	7.50
		2400	97.05 ± 4.80	4.95	95.50 ± 6.23	6.52
	Heart	5	111.27 ± 7.32	6.58	107.46 ± 10.58	9.85
		8	99.28 ± 5.48	5.52	101.15 ± 11.46	11.33
		140	93.45 ± 4.34	4.65	93.19 ± 5.33	5.72
		2400	105.92 ± 9.54	9.01	104.46 ± 6.66	6.38
	Liver	5	100.90 ± 5.40	5.36	108.30 ± 7.82	7.22
		8	99.47 ± 2.82	2.84	100.52 ± 5.00	4.98
		140	88.60 ± 2.60	2.93	93.53 ± 6.33	6.77
		2400	102.50 ± 1.52	1.48	101.42 ± 2.46	2.42
	Spleen	5	111.00 ± 6.99	6.29	104.42 ± 9.31	8.91
		8	98.90 ± 3.55	3.59	100.74 ± 6.77	6.72
		140	96.07 ± 3.95	4.11	93.72 ± 5.30	5.66
		2400	100.02 ± 3.39	3.39	103.13 ± 5.06	4.91
	Lung	5	113.83 ± 4.07	3.58	113.44 ± 5.37	4.73
		8	103.28 ± 10.46	10.13	102.03 ± 10.16	9.96
		140	104.17 ± 3.76	3.61	103.83 ± 4.05	3.90
		2400	107.83 ± 4.07	3.77	107.06 ± 5.02	4.69
	Kidney	5	100.70 ± 4.28	4.25	103.81 ± 6.61	6.36
		8	98.38 ± 5.31	5.40	99.18 ± 4.36	4.39
		140	95.37 ± 4.19	4.39	100.44 ± 7.07	7.04
		2400	106.80 ± 9.74	9.12	104.87 ± 6.46	6.16
	Small intestine	5	109.98 ± 10.97	9.97	105.41 ± 10.19	9.67
		8	99.30 ± 8.94	9.01	100.44 ± 9.18	9.14
		140	88.73 ± 8.96	10.10	86.31 ± 7.66	8.88
		2400	103.30 ± 6.82	6.60	98.26 ± 7.60	7.73
	Stomach	5	109.83 ± 7.05	6.42	102.67 ± 10.09	9.83
		8	101.58 ± 2.96	2.92	95.73 ± 8.07	8.43
		140	94.88 ± 2.74	2.88	92.09 ± 5.17	5.62
		2400	113.17 ± 4.88	4.31	108.96 ± 7.21	6.62
	Brain	5	105.75 ± 10.81	10.22	105.82 ± 8.77	8.29
		8	104.78 ± 7.46	7.12	100.57 ± 8.11	8.06
		140	101.22 ± 4.59	4.53	98.93 ± 5.73	5.80
		2400	113.83 ± 3.31	2.91	111.39 ± 5.54	4.98

Khaephuoside A	Plasma	5	101.63 ± 9.93	9.77	105.66 ± 10.50	9.94
		8	101.02 ± 9.64	9.55	101.78 ± 7.85	7.71
		120	112.33 ± 4.76	4.24	106.16 ± 6.11	5.76
		1800	104.08 ± 4.39	4.21	102.52 ± 4.41	4.31
	Heart	5	113.50 ± 9.18	8.09	103.33 ± 10.92	10.57
		8	100.82 ± 6.19	6.14	98.68 ± 7.06	7.16
		120	96.73 ± 2.92	3.02	89.72 ± 8.01	8.93
		1800	109.12 ± 9.45	8.66	102.43 ± 8.13	7.94
	Liver	5	99.97 ± 7.06	7.07	101.51 ± 8.49	8.36
		8	104.10 ± 5.07	4.87	101.81 ± 4.02	3.95
		120	99.33 ± 0.62	0.62	100.43 ± 4.55	4.53
		1800	108.00 ± 6.10	5.65	103.94 ± 5.60	5.39
	Spleen	5	98.73 ± 11.21	11.35	103.62 ± 9.47	9.14
		8	100.98 ± 6.25	6.19	103.08 ± 6.75	6.55
		120	99.35 ± 2.52	2.54	109.89 ± 8.84	8.04
		1800	109.00 ± 4.60	4.22	113.06 ± 5.36	4.74
	Lung	5	108.17 ± 10.19	9.42	109.93 ± 7.6	6.92
		8	109.90 ± 11.25	10.24	106.91 ± 9.95	9.31
		120	90.32 ± 6.12	6.78	95.54 ± 6.53	6.83
		1800	100.73 ± 2.02	2.00	103.65 ± 6.91	6.66
	Kidney	5	106.82 ± 7.91	7.40	109.94 ± 6.29	5.72
		8	113.67 ± 2.94	2.59	111.40 ± 6.12	5.49
		120	105.30 ± 6.14	5.83	107.78 ± 6.91	6.41
		1800	105.02 ± 9.81	9.34	108.84 ± 7.60	6.99
	Small intestine	5	106.50 ± 8.53	8.01	104.08 ± 10.11	9.71
		8	99.98 ± 9.43	9.13	101.89 ± 11.58	11.36
		120	89.90 ± 6.84	7.61	93.74 ± 7.80	8.32
		1800	104.45 ± 6.22	5.96	105.44 ± 5.15	4.89
	Stomach	5	96.63 ± 9.15	9.47	95.98 ± 10.26	10.69
		8	90.92 ± 7.96	8.76	97.66 ± 8.61	8.82
		120	104.00 ± 6.13	5.90	103.24 ± 5.31	5.15
		1800	109.83 ± 4.83	4.40	110.50 ± 4.54	4.11
	Brain	5	108.90 ± 9.74	8.95	107.11 ± 7.94	7.41
		8	108.55 ± 9.90	9.12	107.23 ± 8.01	7.47
		120	113.17 ± 6.71	5.93	110.13 ± 6.09	5.53
		1800	100.50 ± 3.99	3.97	108.44 ± 7.63	7.03

3,4-Dimethoxyphenyl-1-O-*β*-apiofuroseyl(1 ⟶ 2)-*β*-D-glucopyranoside	Plasma	25	109.00 ± 4.05	3.72	101.34 ± 9.10	8.98
		50	105.82 ± 10.95	10.35	102.61 ± 7.89	7.69
		350	109.83 ± 5.23	4.76	104.49 ± 8.60	8.23
		2400	96.82 ± 2.76	2.85	100.71 ± 7.50	7.45
	Heart	25	99.12 ± 5.06	5.10	96.73 ± 8.08	8.35
		50	96.45 ± 6.75	7.00	98.20 ± 8.28	8.43
		350	89.15 ± 4.83	5.42	90.19 ± 6.82	7.56
		2400	99.80 ± 10.89	10.91	99.33 ± 7.66	7.71
	Liver	25	105.07 ± 6.62	6.30	103.78 ± 6.39	6.16
		50	108.48 ± 7.39	6.82	108.22 ± 7.38	6.82
		350	112.15 ± 6.89	6.14	113.99 ± 4.83	4.24
		2400	98.28 ± 3.67	3.73	100.28 ± 3.24	3.23
	Spleen	25	102.03 ± 10.68	10.47	104.78 ± 8.63	8.23
		50	99.90 ± 520	5.21	103.82 ± 7.77	7.48
		350	97.70 ± 4.12	4.21	109.12 ± 9.07	8.31
		2400	100.65 ± 5.46	5.43	110.22 ± 8.94	8.11
	Lung	25	96.17 ± 7.59	7.89	99.80 ± 10.99	11.01
		50	106.78 ± 7.08	6.63	107.49 ± 8.16	7.59
		350	96.42 ± 4.49	4.66	100.05 ± 5.50	5.49
		2400	99.67 ± 6.29	6.31	99.32 ± 5.68	5.72
	Kidney	25	97.02 ± 9.75	10.05	96.23 ± 9.38	9.74
		50	108.00 ± 5.66	5.24	106.69 ± 7.00	6.56
		350	99.63 ± 5.83	5.85	100.46 ± 6.78	6.75
		2400	96.50 ± 7.41	7.68	97.74 ± 6.31	6.46
	Small intestine	25	98.25 ± 8.92	9.08	97.96 ± 7.73	7.90
		50	101.58 ± 6.41	6.31	101.89 ± 7.13	7.00
		350	89.58 ± 4.27	4.76	98.17 ± 8.39	8.55
		2400	100.18 ± 4.77	4.76	101.32 ± 4.83	4.77
	Stomach	25	103.63 ± 5.21	5.02	99.07 ± 9.64	9.73
		50	101.90 ± 9.32	9.14	97.96 ± 9.33	9.52
		350	114.17 ± 3.19	2.79	109.34 ± 6.14	5.62
		2400	100.62 ± 3.42	3.40	99.42 ± 4.66	4.69
	Brain	25	106.95 ± 12.13	11.34	99.13 ± 11.26	11.36
		50	105.78 ± 7.63	7.22	107.10 ± 7.86	7.34
		350	110.00 ± 9.01	8.19	112.56 ± 6.93	6.16
		2400	99.42 ± 2.90	2.91	101.13 ± 5.44	5.38

**Table 4:** Summary of extraction recovery and matrix effect of four analytes in plasma and tissue samples (*n* = 6).

Compounds	Matrix	Concentration (ng/mL)	Matrix effect (%)	RSD (%)	Extraction recovery (%)	RSD (%)
Chlorogenic acid	Plasma	2	97.26 ± 5.35	5.50	80.45 ± 4.36	5.42
		4	109.75 ± 4.40	4.01	80.04 ± 3.08	3.85
		100	82.80 ± 6.51	7.86	69.49 ± 4.05	5.84
		2400	91.66 ± 1.53	1.67	72.26 ± 1.16	1.61
	Heart	2	116.54 ± 3.03	2.60	80.53 ± 9.62	11.95
		4	98.55 ± 2.71	2.75	76.82 ± 4.77	6.21
		100	97.78 ± 1.76	1.80	62.02 ± 2.24	3.61
		2400	93.25 ± 0.76	0.81	63.32 ± 0.58	0.91
	Liver	2	83.69 ± 4.24	5.06	97.06 ± 3.75	3.86
		4	79.96 ± 4.79	5.94	79.07 ± 2.90	3.66
		100	63.82 ± 2.51	3.94	66.44 ± 0.24	0.37
		2400	80.01 ± 0.90	1.12	69.83 ± 1.55	2.22
	Spleen	2	99.89 ± 2.61	2.61	80.09 ± 1.22	1.52
		4	100.56 ± 1.99	1.98	86.82 ± 2.88	3.32
		100	92.05 ± 1.01	1.10	67.29 ± 1.81	2.69
		2400	91.66 ± 0.81	0.88	73.12 ± 1.61	2.20
	Lung	2	104.73 ± 1.78	1.70	81.80 ± 2.52	3.08
		4	99.34 ± 2.07	2.09	88.65 ± 1.55	1.75
		100	98.65 ± 1.24	1.25	80.24 ± 2.11	2.63
		2400	96.68 ± 1.81	1.88	76.89 ± 1.69	2.20
	Kidney	2	90.49 ± 5.66	6.26	78.34 ± 4.02	5.13
		4	98.52 ± 4.21	4.27	86.92 ± 3.00	3.46
		100	95.03 ± 5.94	6.25	70.73 ± 4.90	6.92
		2400	89.55 ± 2.38	3.16	72.70 ± 1.50	2.07
	Small intestine	2	56.96 ± 6.16	10.81	56.20 ± 3.36	5.98
		4	63.67 ± 2.46	4.14	51.85 ± 1.94	3.73
		100	53.67 ± 1.06	1.97	61.26 ± 1.87	3.05
		2400	50.17 ± 1.72	3.42	62.66 ± 1.06	1.70
	Stomach	2	114.40 ± 5.01	4.46	85.33 ± 5.20	6.10
		4	116.62 ± 8.80	7.55	91.66 ± 6.11	6.67
		100	102.87 ± 3.24	3.15	66.48 ± 3.94	5.93
		2400	98.38 ± 1.63	1.65	69.41 ± 1.56	2.25
	Brain	2	97.89 ± 6.42	6.56	82.33 ± 4.65	5.65
		4	105.09 ± 2.99	2.84	75.80 ± 3.59	4.74
		100	90.02 ± 5.46	6.06	71.37 ± 1.17	1.63
		2400	90.56 ± 2.16	2.39	86.06 ± 1.68	1.95

Naucleactonin C	Plasma	5	104.57 ± 6.94	6.64	100.30 ± 11.77	11.74
		8	102.54 ± 2.55	2.48	106.68 ± 6.31	5.91
		140	104.07 ± 2.88	2.77	96.41 ± 1.66	1.72
		2400	102.64 ± 2.09	2.04	88.27 ± 1.05	1.19
	Heart	5	98.86 ± 2.67	2.70	97.71 ± 1.95	2.00
		8	99.69 ± 3.96	3.97	96.33 ± 7.19	7.46
		140	106.24 ± 4.87	4.58	85.37 ± 2.90	3.40
		2400	99.81 ± 1.48	1.48	86.76 ± 1.61	1.86
	Liver	5	101.81 ± 10.88	10.69	92.06 ± 9.87	10.72
		8	111.16 ± 3.12	2.81	85.03 ± 4.91	5.77
		140	97.92 ± 5.09	5.20	84.88 ± 4.52	5.33
		2400	97.02 ± 1.11	1.14	80.54 ± 0.83	1.03
	Spleen	5	90.83 ± 3.65	4.02	100.21 ± 9.21	9.19
		8	93.78 ± 6.07	6.47	99.90 ± 5.49	5.50
		140	97.28 ± 2.71	2.79	84.00 ± 1.54	1.83
		2400	100.67 ± 0.79	0.78	84.60 ± 1.40	1.65
	Lung	5	98.17 ± 7.63	7.77	105.63 ± 3.87	3.66
		8	115.99 ± 7.97	5.01	99.21 ± 12.43	12.53
		140	98.73 ± 1.39	1.41	95.48 ± 2.88	3.01
		2400	99.21 ± 0.91	0.92	84.40 ± 1.82	2.15
	Kidney	5	99.90 ± 5.10	5.10	94.08 ± 8.11	8.62
		8	100.35 ± 5.14	5.12	105.08 ± 5.22	4.97
		140	101.18 ± 0.70	0.69	86.02 ± 4.39	5.10
		2400	102.49 ± 1.87	1.83	86.64 ± 2.26	2.60
	Small intestine	5	98.00 ± 5.51	5.62	98.55 ± 9.61	9.75
		8	99.37 ± 8.48	8.90	82.00 ± 5.03	6.14
		140	95.42 ± 5.52	5.79	91.73 ± 1.67	1.82
		2400	86.75 ± 2.24	2.58	86.61 ± 3.15	3.63
	Stomach	5	101.53 ± 3.55	3.50	97.93 ± 4.19	4.28
		8	101.21 ± 3.87	3.82	99.25 ± 3.17	3.20
		140	99.62 ± 1.19	1.20	82.46 ± 1.88	2.28
		2400	101.37 ± 1.35	1.33	75.99 ± 2.77	3.64
	Brain	5	97.50 ± 6.50	6.67	88.08 ± 3.12	3.54
		8	105.47 ± 5.14	4.87	88.38 ± 7.32	8.29
		140	102.02 ± 0.85	0.83	97.63 ± 4.35	4.46
		2400	101.24 ± 2.44	2.41	99.47 ± 1.42	1.43

Khaephuoside A	Plasma	5	64.06 ± 5.66	8.84	88.84 ± 5.10	5.75
		8	65.86 ± 5.65	8.57	92.35 ± 8.73	9.45
		120	55.55 ± 4.05	7.29	85.88 ± 2.06	2.40
		1800	58.01 ± 1.93	3.33	85.24 ± 3.30	3.87
	Heart	5	70.92 ± 8.09	11.41	96.07 ± 5.37	5.59
		8	77.13 ± 3.56	4.61	82.29 ± 3.95	4.80
		120	54.79 ± 1.14	2.08	92.18 ± 1.64	1.78
		1800	59.93 ± 4.64	7.75	86.98 ± 4.87	5.59
	Liver	5	73.90 ± 6.84	9.26	105.35 ± 11.02	10.46
		8	53.20 ± 1.59	2.98	103.34 ± 3.75	3.63
		120	58.17 ± 1.12	1.93	77.46 ± 2.17	2.80
		1800	61.29 ± 2.80	4.58	84.49 ± 1.70	2.01
	Spleen	5	68.33 ± 1.41	2.07	90.99 ± 7.89	8.67
		8	60.36 ± 2.58	4.28	96.38 ± 2.77	2.88
		120	58.12 ± 0.42	0.73	90.31 ± 2.80	3.10
		1800	61.44 ± 1.13	1.84	86.75 ± 0.98	1.13
	Lung	5	84.39 ± 4.20	4.97	77.71 ± 3.73	4.81
		8	72.06 ± 6.89	9.56	87.06 ± 7.30	8.38
		120	59.00 ± 2.55	4.33	86.34 ± 3.48	4.03
		1800	61.35 ± 4.41	7.19	79.39 ± 6.48	8.17
	Kidney	5	57.46 ± 1.80	3.13	97.31 ± 4.38	4.50
		8	51.41 ± 2.91	5.66	99.78 ± 3.41	3.42
		120	45.79 ± 1.30	2.84	99.65 ± 1.23	1.23
		1800	47.23 ± 1.38	2.93	101.56 ± 2.17	2.14
	Small intestine	5	56.14 ± 1.47	2.61	102.93 ± 10.44	10.15
		8	67.00 ± 5.43	8.10	94.63 ± 6.27	6.63
		120	62.69 ± 2.06	3.29	100.39 ± 5.00	4.98
		1800	71.51 ± 2.08	2.91	99.78 ± 1.06	1.06
	Stomach	5	56.84 ± 3.21	5.64	95.49 ± 6.60	6.91
		8	63.57 ± 5.59	8.80	83.81 ± 6.89	8.22
		120	54.53 ± 2.44	4.48	77.49 ± 4.08	5.27
		1800	55.57 ± 1.07	1.92	79.96 ± 0.87	1.09
	Brain	5	76.07 ± 1.69	2.22	93.48 ± 8.70	9.30
		8	76.50 ± 6.23	8.15	102.39 ± 6.80	6.64
		120	87.44 ± 3.23	3.70	90.50 ± 0.99	1.09
		1800	79.81 ± 2.15	2.70	100.48 ± 1.42	1.41

3,4-Dimethoxyphenyl-1-O-*β*-apiofuroseyl(1 ⟶ 2)-*β*-D-glucopyranoside	Plasma	25	64.80 ± 4.95	7.64	88.62 ± 8.56	9.66
		50	65.25 ± 0.81	1.24	89.23 ± 4.24	4.76
		350	60.35 ± 3.67	6.08	85.65 ± 1.17	1.37
		2400	55.98 ± 2.34	4.18	84.82 ± 3.16	3.72
	Heart	25	79.81 ± 2.39	2.99	80.56 ± 4.05	5.03
		50	73.97 ± 1.84	2.49	89.91 ± 2.89	3.21
		350	69.51 ± 4.04	5.81	85.34 ± 4.20	4.92
		2400	64.96 ± 2.28	3.51	85.00 ± 3.15	3.71
	Liver	25	55.02 ± 4.02	7.31	88.70 ± 8.38	9.45
		50	53.00 ± 1.63	3.08	94.52 ± 4.76	5.04
		350	52.69 ± 2.12	4.03	83.51 ± 2.63	3.15
		2400	51.60 ± 1.44	2.80	87.94 ± 2.88	3.27
	Spleen	25	81.22 ± 3.27	4.02	98.19 ± 2.87	2.92
		50	87.52 ± 4.51	5.16	92.02 ± 3.15	3.42
		350	81.89 ± 1.03	1.26	89.66 ± 2.42	2.70
		2400	80.06 ± 3.07	3.83	80.48 ± 2.15	2.68
	Lung	25	91.11 ± 4.01	4.40	68.36 ± 4.42	6.47
		50	92.34 ± 3.46	3.75	77.05 ± 2.14	2.78
		350	77.28 ± 4.36	5.65	83.53 ± 3.42	4.09
		2400	76.69 ± 2.73	3.56	75.92 ± 0.73	0.97
	Kidney	25	63.39 ± 1.54	2.43	86.96 ± 4.51	5.19
		50	64.43 ± 1.53	2.38	99.12 ± 2.65	2.67
		350	53.76 ± 3.07	5.72	101.00 ± 1.81	1.79
		2400	60.75 ± 1.53	2.51	103.75 ± 3.17	3.05
	Small intestine	25	64.61 ± 4.23	6.55	98.39 ± 11.15	11.33
		50	68.08 ± 3.68	5.40	90.4 ± 3.02	3.34
		350	70.33 ± 6.13	8.71	101.86 ± 5.70	5.60
		2400	64.51 ± 2.35	3.64	99.20 ± 1.01	1.02
	Stomach	25	68.84 ± 1.18	1.72	92.61 ± 3.13	3.38
		50	62.45 ± 2.20	3.52	88.53 ± 3.44	3.89
		350	65.27 ± 1.67	2.56	77.56 ± 1.83	2.36
		2400	62.11 ± 0.87	1.40	83.08 ± 2.16	2.60
	Brain	25	71.85 ± 1.85	2.57	90.54 ± 1.48	1.64
		50	78.40 ± 2.29	2.92	86.20 ± 4.87	5.65
		350	85.96 ± 2.91	3.39	93.34 ± 1.88	2.02
		2400	75.94 ± 1.52	2.00	101.87 ± 1.82	1.78

**Table 5:** Summary of stability of four analytes in plasma and tissue samples (*n* = 6).

Compounds	Matrix	Concentration (ng/mL)	Ambient temperature for 4 h		Autosampler at 4°C for 8 h		Three freeze-thraw cycles		−80°C for 30 days	
			Accuracy	RSD (%)	Accuracy	RSD (%)	Accuracy	RSD (%)	Accuracy	RSD (%)
Chlorogenic acid	Plasma	2	101.68 ± 8.39	8.25	98.25 ± 7.83	7.97	101.23 ± 12.44	12.29	93.35 ± 10.78	11.55
		4	86.78 ± 6.16	7.09	97.90 ± 11.88	12.14	95.02 ± 5.39	5.67	98.92 ± 12.32	12.46
		100	112.33 ± 3.72	3.31	111.33 ± 4.37	3.92	100.68 ± 6.22	6.18	113.17 ± 4.96	4.38
		2400	98.40 ± 5.56	5.65	99.47 ± 7.59	7.63	91.98 ± 4.75	5.17	112.67 ± 7.58	6.73
	Heart	2	102.73 ± 12.98	12.64	101.70 ± 10.91	10.72	91.72 ± 6.33	6.90	88.98 ± 5.73	6.43
		4	96.88 ± 11.06	11.42	99.50 ± 6.21	6.24	99.03 ± 10.17	10.27	102.27 ± 10.30	10.07
		100	112.83 ± 9.11	8.07	111.93 ± 11.77	10.52	111.00 ± 7.72	6.96	112.62 ± 8.15	7.24
		2400	108.60 ± 8.41	7.75	101.43 ± 10.34	10.20	110.55 ± 8.11	7.33	104.85 ± 10.69	10.19
	Liver	2	88.47 ± 6.93	7.83	87.82 ± 6.45	7.35	88.47 ± 7.22	8.16	87.43 ± 7.74	8.86
		4	86.27 ± 7.32	8.48	87.33 ± 5.74	6.57	86.00 ± 6.23	7.24	87.30 ± 4.35	4.98
		100	99.82 ± 2.89	2.89	106.33 ± 2.88	2.70	102.27 ± 4.97	4.86	87.13 ± 5.77	6.62
		2400	104.17 ± 2.14	2.05	100.92 ± 3.08	3.05	99.98 ± 1.86	1.86	87.02 ± 5.24	6.03
	Spleen	2	97.62 ± 8.11	8.30	109.83 ± 9.95	9.06	105.55 ± 7.56	7.17	109.70 ± 13.05	11.90
		4	105.82 ± 9.91	9.37	100.15 ± 9.69	9.67	103.68 ± 9.71	9.36	98.88 ± 4.16	4.20
		100	112.00 ± 7.82	6.98	111.12 ± 7.31	6.58	106.82 ± 6.88	6.44	111.17 ± 7.17	6.45
		2400	101.52 ± 7.73	7.61	104.00 ± 7.45	7.17	106.67 ± 5.43	5.09	104.73 ± 7.04	6.72
	Lung	2	103.27 ± 8.01	7.75	111.67 ± 5.43	4.86	112.93 ± 9.57	8.48	113.77 ± 9.20	8.08
		4	101.83 ± 6.41	6.30	100.55 ± 8.58	8.53	100.07 ± 10.39	10.38	98.77 ± 6.15	6.23
		100	111.50 ± 5.47	4.90	110.17 ± 10.93	9.92	110.17 ± 7.14	6.48	110.03 ± 9.33	8.46
		2400	100.67 ± 10.17	10.11	98.78 ± 7.01	7.10	104.88 ± 8.23	7.85	110.17 ± 4.54	4.12
	Kidney	2	110.07 ± 7.34	6.67	88.18 ± 4.98	5.64	97.22 ± 7.67	7.89	89.87 ± 9.23	10.27
		4	87.63 ± 7.76	8.86	88.40 ± 6.19	7.00	87.60 ± 9.26	10.58	89.60 ± 9.05	10.10
		100	112.00 ± 7.01	6.26	111.92 ± 8.86	7.91	111.17 ± 7.44	6.69	103.22 ± 11.32	10.97
		2400	100.55 ± 5.11	5.09	98.10 ± 4.87	4.97	100.45 ± 3.96	3.94	101.23 ± 4.69	4.63
	Small intestine	2	105.00 ± 8.20	7.81	102.63 ± 3.52	3.43	103.90 ± 4.49	4.32	101.42 ± 7.82	7.71
		4	91.73 ± 4.49	4.89	99.47 ± 5.10	5.13	99.78 ± 7.70	7.72	106.70 ± 5.43	5.09
		100	95.90 ± 12.31	12.84	108.67 ± 12.04	11.08	100.93 ± 12.33	12.22	99.07 ± 12.41	12.52
		2400	112.00 ± 5.37	4.79	111.67 ± 7.26	6.50	111.67 ± 6.12	5.48	110.83 ± 6.68	6.02
	Stomach	2	113.08 ± 11.06	9.78	111.67 ± 6.25	5.60	113.58 ± 8.22	7.24	109.08 ± 9.97	9.14
		4	102.70 ± 10.17	9.90	97.82 ± 9.05	9.25	98.87 ± 9.82	9.94	89.87 ± 7.04	7.84
		100	93.75 ± 8.65	9.22	94.82 ± 5.05	5.33	93.47 ± 6.76	7.23	107.33 ± 5.89	5.49
		2400	107.17 ± 8.68	8.10	96.60 ± 3.60	3.72	100.23 ± 4.63	4.62	99.73 ± 10.09	10.12
	Brain	2	110.33 ± 7.55	6.85	106.15 ± 12.36	11.65	105.65 ± 10.92	10.34	90.73 ± 10.25	11.30
		4	89.12 ± 9.00	10.10	87.57 ± 8.47	9.67	90.68 ± 10.51	11.59	105.32 ± 9.72	9.23
		100	91.93 ± 9.93	10.80	109.17 ± 7.44	6.82	108.13 ± 9.91	9.17	105.48 ± 6.92	6.56
		2400	102.93 ± 5.79	5.62	106.72 ± 9.83	9.21	96.57 ± 10.12	10.48	97.98 ± 9.56	9.75

Naucleactonin C	Plasma	5	101.95 ± 10.33	10.14	106.67 ± 10.88	10.20	91.87 ± 8.63	9.39	109.57 ± 12.66	11.55
		8	108.88 ± 1.87	1.72	105.01 ± 6.32	6.02	105.88 ± 1.87	1.77	113.33 ± 5.32	4.69
		140	87.67 ± 9.20	10.50	87.88 ± 4.14	4.71	111.88 ± 1.87	1.67	111.57 ± 9.60	8.60
		2400	88.93 ± 6.01	6.75	89.25 ± 7.58	8.49	89.17 ± 7.49	8.40	113.83 ± 7.14	6.27
	Heart	5	111.97 ± 11.63	10.39	100.78 ± 9.63	9.56	107.98 ± 9.45	8.75	107.42 ± 10.24	9.53
		8	110.50 ± 4.42	4.00	107.25 ± 9.25	8.62	109.08 ± 8.25	7.56	110.87 ± 9.04	8.15
		140	102.78 ± 6.89	6.71	104.88 ± 3.80	3.62	100.40 ± 9.13	9.09	98.28 ± 5.70	5.80
		2400	112.33 ± 10.41	9.26	111.50 ± 5.68	5.10	112.33 ± 5.72	5.09	110.50 ± 4.81	4.35
	Liver	5	101.78 ± 11.83	11.63	107.70 ± 5.36	4.98	98.32 ± 6.66	6.78	98.38 ± 12.07	12.27
		8	87.60 ± 5.90	6.73	86.47 ± 4.52	5.23	90.60 ± 4.91	5.42	86.00 ± 8.60	10.00
		140	88.57 ± 2.44	2.76	95.00 ± 4.15	4.37	96.28 ± 4.36	4.53	97.32 ± 7.17	7.36
		2400	98.93 ± 3.81	3.85	91.37 ± 2.51	2.75	89.35 ± 3.70	4.15	89.97 ± 7.32	8.14
	Spleen	5	110.33 ± 8.71	7.89	112.92 ± 8.72	7.72	110.67 ± 8.57	7.75	109.68 ± 12.57	11.46
		8	99.28 ± 13.31	13.41	93.42 ± 8.00	8.57	96.13 ± 11.98	12.46	101.90 ± 10.25	10.06
		140	93.13 ± 6.10	6.55	95.27 ± 7.08	7.43	89.18 ± 8.23	9.23	92.35 ± 2.70	2.92
		2400	103.33 ± 8.37	8.10	105.67 ± 8.50	8.05	107.73 ± 8.10	7.51	109.83 ± 4.96	4.51
	Lung	5	109.50 ± 11.47	10.48	100.40 ± 10.26	10.22	101.05 ± 5.33	5.28	95.98 ± 10.47	10.91
		8	94.73 ± 8.92	9.41	91.40 ± 9.71	10.63	101.90 ± 10.45	10.25	99.03 ± 10.49	10.59
		140	102.38 ± 4.12	4.03	94.45 ± 5.92	6.27	93.75 ± 8.53	9.09	92.58 ± 3.60	3.89
		2400	111.37 ± 9.27	8.33	111.85 ± 9.53	8.52	112.125 ± 9.66	8.61	111.67 ± 3.39	3.03
	Kidney	5	109.33 ± 7.55	6.91	106.08 ± 8.96	8.44	98.92 ± 9.91	10.02	97.18 ± 10.46	10.76
		8	99.10 ± 4.24	4.27	97.38 ± 7.31	7.51	91.10 ± 9.20	10.10	94.77 ± 7.62	8.04
		140	96.08 ± 7.36	7.66	95.43 ± 7.99	8.37	101.10 ± 4.98	4.92	100.25 ± 8.25	8.23
		2400	108.00 ± 5.62	5.20	103.87 ± 4.68	4.51	104.57 ± 6.09	5.82	102.30 ± 5.90	5.77
	Small intestine	5	111.90 ± 12.07	10.79	111.67 ± 8.14	7.29	111.17 ± 8.33	7.49	112.67 ± 8.78	7.79
		8	103.33 ± 3.39	3.28	100.03 ± 11.46	11.46	100.60 ± 10.24	10.18	104.25 ± 9.49	9.10
		140	88.60 ± 9.48	10.70	89.80 ± 8.30	9.24	87.88 ± 5.57	6.34	90.17 ± 7.26	8.05
		2400	107.48 ± 8.23	7.66	103.75 ± 6.68	6.44	108.43 ± 8.65	7.98	102.32 ± 8.65	8.45
	Stomach	5	111.00 ± 7.90	7.12	112.17 ± 7.25	6.46	113.12 ± 9.97	8.81	113.83 ± 2.99	2.63
		8	107.32 ± 12.96	12.08	98.37 ± 10.75	10.93	105.90 ± 9.13	8.62	103.55 ± 9.49	9.16
		140	88.15 ± 7.91	8.98	86.38 ± 8.38	9.70	87.12 ± 5.24	6.1	88.28 ± 6.31	7.15
		2400	100.53 ± 7.29	7.25	99.52 ± 5.85	5.88	104.90 ± 5.53	5.28	102.32 ± 8.22	8.03
	Brain	5	106.35 ± 11.82	11.11	109.83 ± 6.05	5.51	98.92 ± 10.65	10.77	104.10 ± 10.88	10.45
		8	88.87 ± 10.43	11.74	90.65 ± 6.37	7.03	89.95 ± 9.23	10.26	88.82 ± 10.57	11.90
		140	88.33 ± 6.74	7.63	87.92 ± 8.15	9.27	87.20 ± 4.98	5.71	87.93 ± 5.81	6.61
		2400	99.43 ± 6.38	6.41	112.67 ± 6.62	5.88	109.67 ± 6.44	5.87	109.00 ± 5.22	4.78

Khaephuoside A	Plasma	5	99.10 ± 9.25	9.33	90.97 ± 9.01	9.90	93.93 ± 11.26	11.98	101.18 ± 3.53	3.49
		8	91.38 ± 12.76	13.96	94.88 ± 12.31	12.97	90.67 ± 9.29	10.25	108.17 ± 9.20	8.50
		120	101.35 ± 4.33	4.28	101.32 ± 3.40	3.36	96.75 ± 4.55	4.70	107.30 ± 11.48	10.70
		1800	99.35 ± 6.04	6.08	101.93 ± 6.90	6.77	96.13 ± 4.03	4.19	112.67 ± 7.66	6.80
	Heart	5	107.60 ± 11.23	10.44	104.15 ± 9.92	9.52	110.67 ± 10.82	9.78	107.45 ± 9.35	8.70
		8	112.67 ± 7.23	6.42	111.83 ± 8.23	7.36	111.33 ± 5.92	5.32	101.00 ± 12.05	11.93
		120	111.67 ± 6.22	5.57	112.67 ± 5.89	5.23	108.58 ± 11.51	10.60	113.50 ± 7.20	6.35
		1800	110.83 ± 2.79	2.51	106.58 ± 8.77	8.22	113.67 ± 4.27	3.76	101.90 ± 12.30	12.07
	Liver	5	93.87 ± 9.31	9.92	106.17 ± 4.88	4.59	95.62 ± 9.82	10.27	100.87 ± 10.27	10.19
		8	108.95 ± 10.77	9.88	101.97 ± 7.93	7.78	105.20 ± 10.21	9.70	91.12 ± 10.79	11.84
		120	107.67 ± 6.22	5.78	111.50 ± 7.26	6.51	101.62 ± 9.62	9.47	104.23 ± 8.68	8.33
		1800	108.83 ± 2.32	2.13	109.83 ± 5.38	4.90	103.20 ± 7.52	7.29	108.93 ± 9.52	8.74
	Spleen	5	110.30 ± 12.31	11.16	112.17 ± 7.94	7.07	111.00 ± 10.26	9.24	111.50 ± 8.41	7.54
		8	112.17 ± 5.64	5.02	111.67 ± 6.19	5.54	111.38 ± 7.36	6.58	110.67 ± 7.17	6.48
		120	107.85 ± 7.83	7.26	105.45 ± 7.95	7.54	98.83 ± 7.51	7.60	105.42 ± 10.79	10.24
		1800	109.77 ± 8.61	7.85	111.17 ± 8.18	7.36	113.17 ± 3.31	2.93	111.83 ± 7.57	6.77
	Lung	5	113.33 ± 8.71	7.69	106.65 ± 12.43	11.65	98.87 ± 9.02	9.12	102.98 ± 9.50	9.23
		8	105.32 ± 7.81	7.41	103.18 ± 10.97	10.63	105.03 ± 9.86	9.38	104.00 ± 11.73	11.28
		120	101.27 ± 3.53	3.49	99.07 ± 11.01	11.11	102.02 ± 6.36	6.23	110.83 ± 5.19	4.69
		1800	110.83 ± 8.23	7.43	112.80 ± 7.46	6.61	110.00 ± 8.88	8.07	112.50 ± 6.80	6.05
	Kidney	5	96.42 ± 6.24	6.47	99.65 ± 10.53	10.57	101.62 ± 11.37	11.19	112.78 ± 11.43	10.13
		8	113.17 ± 6.46	5.71	110.83 ± 7.88	7.11	113.00 ± 6.99	6.18	113.17 ± 7.19	6.36
		120	111.67 ± 5.32	4.76	111.83 ± 5.31	4.75	112.00 ± 5.93	5.30	112.00 ± 5.62	5.02
		1800	102.03 ± 4.44	4.35	97.37 ± 6.84	7.02	100.50 ± 5.53	5.50	101.12 ± 6.56	6.48
	Small intestine	5	110.02 ± 8.19	7.44	112.83 ± 3.43	3.04	100.88 ± 9.39	9.30	94.98 ± 9.42	9.91
		8	105.62 ± 9.76	9.24	109.53 ± 10.14	9.25	101.00 ± 10.42	10.31	107.70 ± 11.47	10.65
		120	103.30 ± 7.75	7.50	106.00 ± 7.29	6.88	102.80 ± 6.09	5.93	101.58 ± 6.26	6.16
		1800	113.00 ± 6.13	5.43	110.83 ± 8.13	7.34	113.67 ± 8.19	7.20	113.12 ± 12.47	11.02
	Stomach	5	113.68 ± 8.42	7.41	112.13 ± 10.23	9.12	111.12 ± 10.43	9.38	110.67 ± 6.09	5.50
		8	111.67 ± 6.59	5.90	111.00 ± 5.22	4.70	109.67 ± 3.33	3.03	104.50 ± 4.71	4.51
		120	113.17 ± 3.66	3.23	111.50 ± 5.86	5.25	112.17 ± 6.15	5.48	110.92 ± 8.36	7.53
		1800	109.83 ± 7.36	6.70	111.92 ± 10.99	9.82	110.50 ± 6.28	5.69	113.83 ± 5.56	4.89
	Brain	5	109.82 ± 10.26	9.34	106.90 ± 12.09	11.31	103.05 ± 9.12	8.85	112.50 ± 5.58	4.96
		8	111.00 ± 7.42	6.52	112.50 ± 7.06	6.28	113.33 ± 3.83	3.38	111.33 ± 6.09	5.47
		120	112.50 ± 5.13	4.56	112.33 ± 6.47	5.76	113.17 ± 5.31	4.69	110.17 ± 8.28	7.52
		1800	105.82 ± 8.31	7.86	112.83 ± 7.68	6.81	110.83 ± 7.52	6.79	113.83 ± 4.49	3.95

3,4-Dimethoxyphenyl-1-O-*β*-apiofuroseyl(1 ⟶ 2)-*β*-D-glucopyranoside	Plasma	25	86.25 ± 5.40	6.26	94.95 ± 9.80	10.32	102.87 ± 10.37	10.08	105.60 ± 10.19	9.65
		50	87.20 ± 7.91	9.07	98.98 ± 11.30	11.30	94.53 ± 9.84	10.41	107.88 ± 14.96	13.87
		350	112.83 ± 4.58	4.06	112.67 ± 6.62	5.88	108.33 ± 5.13	4.73	109.07 ± 1.87	1.72
		2400	89.98 ± 4.76	5.29	89.43 ± 7.42	8.30	86.07 ± 3.39	3.93	111.74 ± 3.76	3.37
	Heart	25	98.10 ± 9.43	9.61	100.45 ± 10.89	10.85	97.55 ± 9.01	9.24	88.38 ± 5.69	6.44
		50	94.60 ± 11.14	7.80	102.70 ± 7.98	7.77	97.05 ± 8.42	8.68	99.12 ± 9.56	9.65
		350	102.57 ± 4.92	4.80	112.63 ± 98.93	7.93	110.33 ± 5.82	5.27	108.38 ± 7.34	6.77
		2400	102.23 ± 6.64	6.49	92.98 ± 8.87	9.54	101.57 ± 6.73	6.63	99.57 ± 9.46	9.50
	Liver	25	91.10 ± 10.37	11.39	102.88 ± 10.85	10.54	97.32 ± 10.44	10.73	104.70 ± 11.97	11.43
		50	105.60 ± 9.04	8.56	112.50 ± 5.65	5.02	107.48 ± 10.54	9.80	95.25 ± 11.29	11.86
		350	97.50 ± 6.63	6.80	113.00 ± 4.00	3.54	103.12 ± 5.26	5.10	108.67 ± 8.98	8.27
		2400	104.32 ± 10.18	9.76	100.42 ± 6.85	6.83	96.25 ± 5.74	5.96	107.68 ± 7.73	7.18
	Spleen	25	88.72 ± 9.93	11.19	104.47 ± 9.00	8.62	90.93 ± 9.15	10.07	112.83 ± 3.60	3.19
		50	113.17 ± 10.59	9.36	108.35 ± 10.58	9.77	95.62 ± 10.90	11.40	107.68 ± 10.39	9.65
		350	111.33 ± 7.63	6.86	111.68 ± 9.21	8.25	112.17 ± 7.99	7.12	112.17 ± 8.40	7.49
		2400	102.27 ± 11.50	11.24	112.00 ± 8.67	7.74	105.32 ± 6.59	6.26	111.00 ± 6.03	5.44
	Lung	25	90.52 ± 10.77	11.90	96.80 ± 11.29	11.67	97.07 ± 10.87	11.20	97.92 ± 11.34	11.59
		50	112.67 ± 7.84	6.96	111.02 ± 7.81	7.04	100.02 ± 12.46	12.46	109.25 ± 11.46	10.49
		350	111.33 ± 6.89	6.19	109.47 ± 11.10	10.14	103.68 ± 11.62	11.21	112.33 ± 6.92	6.16
		2400	98.85 ± 9.83	9.94	101.82 ± 8.67	8.52	100.62 ± 7.71	7.66	109.12 ± 10.69	9.79
	Kidney	25	100.05 ± 11.92	11.92	101.42 ± 8.63	8.51	96.23 ± 9.14	9.50	110.83 ± 6.24	5.63
		50	98.37 ± 7.53	7.65	102.72 ± 10.29	10.02	106.75 ± 9.15	8.57	99.88 ± 9.65	9.66
		350	90.05 ± 8.47	9.40	102.03 ± 4.61	4.52	111.83 ± 7.03	6.28	105.40 ± 10.28	9.75
		2400	104.72 ± 8.96	8.56	106.27 ± 10.19	9.59	107.85 ± 9.16	8.49	104.82 ± 8.59	8.20
	Small intestine	25	87.47 ± 7.17	8.20	96.42 ± 9.13	9.47	94.10 ± 8.05	8.55	99.25 ± 6.06	6.11
		50	109.83 ± 2.56	2.33	100.75 ± 6.11	6.07	106.83 ± 5.34	5.00	101.80 ± 8.78	8.62
		350	111.17 ± 7.73	6.95	113.67 ± 8.76	7.70	113.00 ± 3.79	3.36	106.33 ± 5.39	5.07
		2400	98.50 ± 5.49	5.57	104.65 ± 7.85	7.50	99.92 ± 5.94	5.94	94.90 ± 8.62	9.08
	Stomach	25	111.83 ± 5.78	5.17	110.50 ± 4.42	4.00	113.00 ± 6.32	5.60	106.32 ± 9.48	8.92
		50	112.75 ± 10.97	9.73	112.67 ± 3.67	3.26	112.83 ± 5.60	4.96	109.83 ± 8.66	7.88
		350	113.17 ± 3.87	3.42	108.17 ± 9.68	8.95	111.00 ± 6.20	5.58	111.83 ± 5.98	5.35
		2400	101.27 ± 7.89	7.80	94.40 ± 6.00	6.35	96.82 ± 8.08	8.34	103.08 ± 9.60	9.32
	Brain	25	99.82 ± 9.82	9.84	107.80 ± 12.79	11.86	100.68 ± 9.80	9.73	105.37 ± 7.31	6.93
		50	109.35 ± 12.15	11.11	112.83 ± 4.26	3.78	113.77 ± 10.36	9.11	113.83 ± 3.87	3.40
		350	111.50 ± 8.64	7.75	110.83 ± 7.47	6.74	111.17 ± 7.63	6.27	112.83 ± 7.78	6.90
		2400	98.95 ± 3.97	4.01	100.10 ± 9.43	9.42	98.23 ± 5.39	5.49	101.50 ± 9.94	9.80

**Table 6:** Summary of primary pharmacokinetic parameters of four analytes (*n* = 6).

Parameters	Unit	Chlorogenic acid (mean ± SD)	Naucleactonin C (mean ± SD)	Khaephuoside A (mean ± SD)	3,4-dimethoxyphenyl-1-O-*β*-apiofuroseyl(1 ⟶ 2)-*β*-D-glucopyranoside (mean ± SD)
AUC (0 − *t*)	*μ*g/L∗h	482.76 ± 124.95	5281.36 ± 440.02	238.70 ± 46.64	212.99 ± 49.29
AUC (0 − ∞)	*μ*g/L∗h	554.37 ± 166.14	5405.02 ± 416.99	262.16 ± 32.24	440.17 ± 216.99
MRT (0 − *t*)	h	5.33 ± 0.65	4.76 ± 1.57	3.19 ± 0.24	1.6 ± 0.14
*t*1/2	h	10.64 ± 6.21	4.59 ± 1.65	3.12 ± 1.70	3.99 ± 2.87
*T *_max_	h	0.25 ± 0.00	1.67 ± 0.52	0.29 ± 0.10	0.33 ± 0.13
CL	L/h/kg	62.94 ± 16.00	0.15 ± 0.01	54.40 ± 6.04	28.63 ± 13.64
*C *_max_	*μ*g/L	242.42 ± 84.67	1008.58 ± 368.84	106.18 ± 23.30	144.13 ± 35.59
Vz/F	L/kg	915.76 ± 451.75	0.966 ± 0.359	253.289 ± 158.11	127.43 ± 65.93

Abbreviations. AUC, area under the plasma concentration–time curve; MRT, mean residence time; *t*1/2, elimination half-life; *T*_max_, time to Cmax; CL, clearance rate; *C*_max_, maximum plasma concentration; Vz/F, apparent distribution volume.

## Data Availability

The data used to support the findings of this study are included within the article and the supplementary information files.

## Supplementary Materials

Supplementary Materials See Figures S1–S2 in the *Supplementary Material* for typical chromatograms of four analytes and IS in liver tissue and kidney tissue.
