# Supplementary material for: Measurement of Pharmacokinetics and Tissue Distribution of Four Compounds from Nauclea officinalis in Rat Plasma and Tissues through HPLC-MS/MS
Source: J Anal Methods Chem. 2022 Dec 21;2022:5297603. doi: 10.1155/2022/5297603 (PMC9797307; doi:10.1155/2022/5297603)
Supplement: Supplementary Materials — See Figures S1–S2 in the Supplementary Material for typical chromatograms of four analytes and IS in liver tissue and kidney tissue. [file 5297603.f1.docx]

FIGURE S1: Typical chromatograms of four analyte and IS in rat liver tissue: A: blank liver homogenate samples, B: blank liver homogenate samples spiked with four analytes (LLOQ) and IS, C: rat liver homogenate samples at 0.5 h after oral administration the *Nauclea officinalis* extracts spiked with IS; 1. Chlorogenic acid, 2. Naucleactonin C, 3. Khaephuoside A, 4. 3,4-dimethoxyphenyl-1-O-*β*-apiofuroseyl(1→2)-*β*-D-glucopyranoside, 5. Chloramphenicol(IS).





FIGURE S2: Typical chromatograms of four analyte and IS in rat kidney tissue: A: blank kidney homogenate samples, B: blank kidney homogenate samples spiked with four analytes (LLOQ) and IS, C: rat kidney homogenate samples at 0.5 h after oral administration the *Nauclea officinalis* extracts spiked with IS; 1. Chlorogenic acid, 2. Naucleactonin C, 3. Khaephuoside A, 4. 3,4-dimethoxyphenyl-1-O-*β*-apiofuroseyl(1→2)-*β*-D-glucopyranoside, 5. Chloramphenicol(IS).
